# High Antibody Responses against *Plasmodium falciparum* in Immigrants after Extended Periods of Interrupted Exposure to Malaria

**DOI:** 10.1371/journal.pone.0073624

**Published:** 2013-08-14

**Authors:** Gemma Moncunill, Alfredo Mayor, Alfons Jiménez, Augusto Nhabomba, Núria Casas-Vila, Laura Puyol, Joseph J. Campo, Maria Nelia Manaca, Ruth Aguilar, María-Jesús Pinazo, Mercè Almirall, Cristina Soler, José Muñoz, Azucena Bardají, Evelina Angov, Sheetij Dutta, Chetan E. Chitnis, Pedro L. Alonso, Joaquim Gascón, Carlota Dobaño

**Affiliations:** 1 Barcelona Centre for International Health Research, (CRESIB, Hospital Clínic-Universitat de Barcelona), Barcelona, Catalonia, Spain; 2 Centro de Investigação em Saúde de Manhiça, Maputo, Mozambique; 3 Hospital Arnau de Vilanova, Lleida, Catalonia, Spain; 4 Hospital Santa Caterina de Salt, Girona, Catalonia, Spain; 5 Walter Reed Army Institute of Research (WRAIR), Silver Spring, Maryland, United States of America; 6 International Centre for Genetic Engineering and Biotechnology (ICGEB), New Delhi, India; Burnet Institute, Australia

## Abstract

**Background:**

Malaria immunity is commonly believed to wane in the absence of *Plasmodium falciparum* exposure, based on limited epidemiological data and short-lived antibody responses in some longitudinal studies in endemic areas.

**Methods:**

A cross-sectional study was conducted among sub-Saharan African adults residing in Spain for 1 up to 38 years (immigrants) with clinical malaria (n=55) or without malaria (n=37), naïve adults (travelers) with a first clinical malaria episode (n=20) and life-long malaria exposed adults from Mozambique (semi-immune adults) without malaria (n=27) or with clinical malaria (n=50). Blood samples were collected and IgG levels against the erythrocytic antigens AMA-1 and MSP-1_42_ (3D7 and FVO strains), EBA-175 and DBL-α were determined by Luminex. IgG levels against antigens on the surface of infected erythrocytes (IEs) were measured by flow cytometry.

**Results:**

Immigrants without malaria had lower IgG levels than healthy semi-immune adults regardless of the antigen tested (P≤0.026), but no correlation was found between IgG levels and time since migration. Upon reinfection, immigrants with malaria had higher levels of IgG against all antigens than immigrants without malaria. However, the magnitude of the response compared to semi-immune adults with malaria depended on the antigen tested. Thus, immigrants had higher IgG levels against AMA-1 and MSP-1_42_ (P≤0.015), similar levels against EBA-175 and DBL-α, and lower levels against IEs (P≤0.016). Immigrants had higher IgG levels against all antigens tested compared to travelers (P≤0.001), both with malaria.

**Conclusions:**

Upon cessation of malaria exposure, IgG responses to malaria-specific antigens were maintained to a large extent, although the conservation and the magnitude of the recall response depended on the nature of the antigen. Studies on immigrant populations can shed light on the factors that determine the duration of malaria specific antibody responses and its effect on protection, with important implications for future vaccine design and public health control measures.

## Introduction

Maintenance of long-term memory responses is critical for achieving protective immunity against many pathogens. The understanding of differential immuno-reactivity to malaria and maintenance of these immune responses is fundamental for the development and design of immunogenic strategies for disease control and eradication. In malaria endemic areas, immunity is acquired gradually with age and continuous exposure, first to severe disease and ultimately to clinical malaria and high parasitemia [1]. Nevertheless, it is thought that upon cessation of exposure to *Plasmodium falciparum* infection immunity wanes rapidly, and this is in contrast with the long-term antibody-mediated immunity that follows one or few exposures to antigens from other infectious microbes [2].

The control of *P. falciparum* infections is complex, and requires the combined action of antibodies (Ab) and cell-mediated immune responses against both pre-erythrocytic and blood stages; and these two effector mechanisms are required for both anti-parasitic as well as clinical immunity [3,4]. The relevance of Ab responses in malaria protection was established several decades ago by immunoglobulin G (IgG) passive transfer experiments [5,6], and different mechanisms of immunity have been proposed. Potential Ab effector actions include: blockade of hepatocyte invasion by sporozoites and red blood cell invasion by merozoites; Ab-dependent cellular killing through interaction of target-bound Ab with certain Fc receptors from cell surfaces; opsonization of infected erythrocytes (IE) inducing phagocytic clearance; and neutralization of the parasite glycosylphosphatidylinositol, inhibiting the induction of the inflammatory cytokine cascade [3]. *P. falciparum* antigens targeted by naturally acquired IgG associated with immunity include the merozoite proteins: apical membrane antigen 1 (AMA-1), the 42-kDa fragment from the C terminus of *merozoite* surface protein 1 (MSP-1_42_), and the 175 kDa erythrocyte binding antigen (EBA-175), all three involved in erythrocyte invasion [7–11]. In addition, variant surface antigens (VSA) expressed on IE membranes are also targets of naturally acquired Ab responses associated with immunity [12]. The *P. falciparum* erythrocyte membrane protein 1 (*Pf*EMP-1) is the major antigen of this VSA family, containing several domains that mediate cytoadherence to host cells, like the Duffy binding-like alpha (DBL-α) domain that is involved in rosetting [13].

Despite the common perception that immune memory to malaria is short-lived in the absence of exposure, most clinical evidence suggests that immunity may last for long periods of time. Immigrants maintain some immunity to clinical malaria, and have milder episodes than naïve adults with a malaria primary infection [14–21]. Also in areas of low or unstable malaria transmission such as Madagascar, adults previously exposed to malaria, even several decades before, were protected against clinical disease during malaria epidemics [22–24].

However, data on the longevity of protective immune responses are scarce. Ab responses to malarial antigens are often thought to be short-lived [25–30], although this has mostly been reported in children in areas where malaria is endemic [27–31]. On the contrary, in adults, long-lived IgG responses have been detected [32–35]. It appears that frequent reinfection is required to maintain high levels of circulating Ab, thus in highly endemic areas Ab levels are stable [36,37], but in low or unstable transmission areas Ab levels diminish quickly after an infection [28,38], showing seasonal variation [26,27,39–41]. Conversely, memory B cells (MBC) can persist with reduced transmission [42–45]. However, a study reported the presence of Ab, but only very low frequencies of malaria-specific MBC in children, suggesting a low induction of malaria-specific circulating MBC as a reason for short-lived anti-malarial Ab responses [46].

The aim of this study was to measure *P. falciparum*-specific Ab responses as indicative of loss or maintenance of immunity in immigrants who have not been exposed to malaria for long periods of time. We compared IgG levels against a panel of blood stage antigens thought to be involved in malaria immunity between previously exposed immigrants, continuously exposed adults and naïve travelers, with or without a clinical malaria episode, using Luminex and flow cytometry.

## Materials and Methods

### Ethics Statement

Written informed consent was obtained from participants before sample collection. Approval for the protocols was obtained from the Hospital Clínic of Barcelona Ethics Review Committee and the National Mozambican Ethics Review Committee. Parasitemic individuals were treated according to standard national guidelines at the time of the studies.

### Study design, subjects and sample collection

Three groups of participants were recruited for this study: (i) sub-Saharan African adults originally from malaria endemic areas residing in Spain (immigrants) without clinical malaria (n=37) or with clinical malaria (n=55) upon return from travel; (ii) adults from a sub-Saharan Africa endemic area with life-long exposure to *P. falciparum* infection (semi-immune adults), with (n=50) or without clinical malaria (n=27); and (iii) naïve adults from a non-endemic area returning from a sub-Saharan Africa malaria endemic region with a first malaria episode (travelers, n=20).

Immigrants were recruited at the Tropical Medicine Units of Hospital Clínic de Barcelona (Barcelona, Spain), Hospital Arnau de Vilanova (Lleida, Spain) and Hospital Santa Caterina de Salt (Girona, Spain) between 2005 and 2009. Travelers were recruited at the Tropical Medicine Unit of the Hospital Clínic de Barcelona (Barcelona, Spain) [47]. Fifty-five immigrants and 20 travelers were diagnosed with *P. falciparum* clinical malaria after traveling to an African country. Clinical malaria was defined as the presence of asexual *P. falciparum* parasites on Giemsa-stained blood smears detected by light microscopy, together with fever. *P. falciparum* parasitemia in blood was measured as the percentage of parasitized red blood cells. Blood samples from acute malaria episodes (day 0) and at convalescence after malaria treatment (day 7) were collected by venipuncture into vacutainers without anticoagulant for serum cryopreservation at -80^°^ C. In addition, blood samples from 37 immigrants visiting the Tropical Medicine Units without malaria were also collected. These immigrants were healthy companions or those presenting with non-malaria diseases. Most of them had a febrile syndrome or traveler diarrhea but the following conditions were also diagnosed: giardiasis, katayama syndrome, mononucleosis syndrome EBV, pneumonia, pruritus eczema, anxiety disorder, appendicitis, dermatitis, toxic syndrome, viral infection, ketoacidosis, diabetes, headache, spontaneous abortion, bacterial lung abscess and HIV infection. Clinical and demographical data were recorded in standardized questionnaires. Data on Ab levels from travelers have been previously published [47], but are re-analysed here for comparison to the immigrant group.

Semi-immune adults were from Manhiça District in Mozambique, where malaria transmission is perennial, with some seasonality and moderate intensity. Fifty semi-immune adults with *P. falciparum* clinical malaria were recruited at the Manhiça District Hospital in the context of a hospital-based study conducted at the Centro de Investigação em Saúde de Manhiça in 2006 [13]. Clinical malaria was defined as the presence of asexual *P. falciparum* parasites on blood smears, together with fever. Blood films were Giemsa-stained, and examined using a light microscope and parasite density in blood was measured as parasites/µL. Additionally, 27 healthy semi-immune adults were recruited in a cross-sectional study in the Manhiça District between 2005 and 2006. They were adults who had not lived in a city and were not parasitemic by microscopy at the moment of collecting the blood samples. Blood samples were collected by venipuncture into heparinized tubes, and plasma samples were cryopreserved at -80^°^ C.

### Recombinant proteins

AMA-1 from the 3D7 strain [48], the receptor-binding region F2 of EBA-175 from the CAMP strain [49] and the DBL-α domain of a *Pf*EMP-1 involved in rosetting [13] were produced as recombinant proteins at ICGEB. AMA-1 from FVO strain, and MSP-1_42_ from 3D7 and FVO strains [50,51] were produced as recombinant proteins at WRAIR.

### Antibody levels to recombinant proteins

IgG responses to *P. falciparum* antigens were determined using Luminex xMAP^TM^ (Luminex Corp., Austin, Texas, USA) and the Bio-Plex 100 platform (Bio-Rad, Hercules, California, USA) as previously described [47,52]. A pool of plasma samples from hyper-immune Mozambican adult volunteers (n=33), and 4 plasma samples from non-exposed European adults (n=42) were added in duplicates to each plate as positive and negative controls, respectively. In addition, curves of a pool of plasma samples from hyper-immune adults were added in each experiment (Figure S1). Study samples were tested in duplicates at the dilutions 1/1,000 and 1/30,000, but only the dilution 1/1,000 was chosen for the statistical analyses because of a wider quantitative dynamic range. In some wells no serum/plasma was added as a control of background. Plates were read using Bio-Plex Manager version 4.0, and at least 100 microspheres per analyte were acquired per sample. Median fluorescent intensity with background fluorescence subtracted was exported, and arbitrary units (AU) concentration for each Ab was calculated by dividing the median fluorescent intensity of each sample by the median fluorescent intensity of the positive control run in each plate.

### Antibodies to IEs surface antigens

Two laboratory clones (CS2, R29), three isolates collected from travelers to endemic regions (IE_Trav1_, IE_Trav2_, IE_Trav3_), two pediatric isolates from Mozambican children, one with uncomplicated malaria (IE_Ch1_) and another with severe malaria (IE_Ch2_), and one isolate from a pregnant Mozambican woman (IE_Woman_), each of whom had the O blood group, were tested for recognition by IgG using flow cytometry as previously described [47]. Cryopreserved ring-stage parasites were thawed in a sorbitol gradient and grown to late trophozoites. Study samples were tested blindly in a single assay against each parasite. A pool of plasma samples from hyper-immune Mozambican adults (n=11) and 10 samples from non-exposed European adults were included as positive and negative controls, respectively. Data from 1,000 positive events was acquired with a Becton-Dickinson (BD) FACSCalibur flow cytometer. Reactivity against IE surface antigens was expressed as the difference between the geometric mean fluorescent intensity (MFI) of IEs and the MFI of uninfected erythrocytes. Representative flow cytometry data are showed in Figure S2.

### Statistical methods

Recognition of recombinant proteins by Ab was considered positive if AU values were above the mean of the negative controls plus 2 standard deviations. Threshold values for seroprevalence were: 238.02 AU for AMA-1 3D7; 1134.73 AU for AMA-1 FVO; 921.18 AU for MSP-1_42_ 3D7; 638.33 AU for MSP-1_42_ FVO; 3110.36 AU for EBA-175; 1572.55 AU for DBL-α. Recognition of parasites by Ab was considered positive if MFI values were above the mean of the negative controls plus 3 standard deviations. Threshold values for seroprevalence were: 36.88 MFI for IE_Trav1_, 38.55 MFI for IE_Trav2_, 24.75 MFI IE_Trav3,_ 14.23 MFI for CS2, 19.92 MFI for R29, 8.69 MFI for E_Ch1,_ 1.98 MFI for IE_Ch2,_ and 5.54 MFI for IE_Woman_. Comparisons between groups for categorical variables were done using χ^2^ test or Fisher’s exact test. Continuous variables were analyzed using the non-parametric Kruskal Wallis test or the Wilcoxon Rank Sum test. Correlations between IgG levels and years since migration were assessed by Spearman’s rank coefficient. All *p*-values were two-sided and considered statistically significant when < 0.05. All data collected were analyzed using Stata version 11.0 (Stata Corporation, College Station, Texas, USA).

## Results

### Characteristics of the study participants

The characteristics of study participants are summarized in Table 1. Immigrants with malaria were originally from Cameroon (n=3, 5.5%), Ghana (n=8, 14.6%), Guinea-Conakry (n=4, 7.3%), Equatorial Guinea (n=12, 21.8%), Gambia (n=8, 14.6%), Mali (n= 4, 7.3%), Mauritania (n=1, 1.8%), Mozambique (n=1, 1.8%), Nigeria (n=6, 10.9%) and Senegal (n=7, 12.7%). Immigrants without malaria were from Benin (n=1, 2.7%), Burkina Faso (n=2, 5.41%), Cameroon (n=3, 8.11%), Guinea-Conakry (n=3, 8.11%), Equatorial Guinea (n=3, 8.11%), Gambia (n=3, 8.11%), Kenya (n=1, 2.7%), Mali (n=9, 24.32%), Mauritania (n=1, 2.7%), Mozambique (n=1, 2.7%), Nigeria (n=3, 8.11%), Senegal (n=5, 13.61%) and Sudan (n=1, 2.7%). Most of the semi-immune adults without malaria were males, whereas there were more females among the semi-immune adults with malaria than in the other groups (*P*=0.001). Visiting countries were very heterogeneous among immigrants and travelers. Immigrants were those returning from visiting their countries of origin. Travelers came from Burkina Faso & Mali & Senegal (n=1, 5.0%), Burkina Faso (n=3, 15.0%), Burkina Faso & Mali & Ghana & Togo (n=1, 5.0%), Ivory Coast (n=1, 5.0%), Guinea-Conakry (n=1, 5.0%), Equatorial Guinea (n=3, 15.0%), Gambia & Senegal (n=1, 5.0%), Madagascar (n=1, 5.0%), Mali (n=1, 5.0%), Mozambique (n=2, 10.0%), Mozambique & South Africa (n=1, 5.0%), Senegal (n=3, 15.0%) and Sierra Leone & Senegal (n=1, 5.0%). Immigrants with malaria had lived a median of 7 years in Spain, whereas immigrants without malaria had lived a median of 5 years (*P*= 0.0362, Table 1). However, 10% of immigrants with malaria were returning from their first trip to their original country, 21% had previously returned 1 to 2 times, 50% had returned 3 to 4 times and 19% had returned more than 5 times. Forty-seven percent of immigrants without malaria had never returned to their original country, 15% had returned 1 to 2 times, 12% had returned 3 to 4 times and 27% had returned more than 5 times. No significant differences were detected in parasitemia between immigrants and travelers (*P*=0.0890, Table 1).

**Table 1:** Characteristics of the study participants.

	**Immigrants**		**Travelers**	**Semi-Immune**	
**Characteristics**	**Malaria**	**No Malaria**	**Malaria**	**Malaria**	**No Malaria**
N					
Day 0	55	37	20	50	27
Day 7	29	0	12	0	0
Age, median IQR (years)^a^	34 (29-; 43)	36 (30; 44)	32 (28.5; 38.5)	33 (27; 42)	41 (39, 43)
Sex^b^, n (%)					
Males	40 (73)	23 (62)	15 (75)	28 (56)	26 (96)
Origin area, n (%)					
Europe	0 (0)	0 (0)	17 (85)	0 (0)	0 (0)
Africa	54 (100)	37 (100)	0 (0)	50 (100)	27 (100)
Others	0 (0)	0 (0)	3 (15)	0 (0)	0 (0)
Time since immigration, median IQR (years)^c^	7 (5; 14)	5 (2; 9)	na	na	na
Number of previous returns, n (%)					
0	5 (10)	16 (47)	na	na	na
1-2	11 (21)	5 (15)	na	na	na
3-4	26 (50)	4 (12)	na	na	na
>5	10 (19)	9 (27)	na	na	na
Parasitemia by microscopy					
median IQR (%)^d^	0.4 (0.02; 1.5)	na	0.075 (0.01;0.8)	nd	na
median IQR (parasites/μl)	nd	na	nd	34783.5 (7721, 55122)	na

Abbreviations: IQR, interquartile range; nd, not determined; na, not applicable

a *P*=0.0515 Kruskal Wallis test. Data missing from 2 semi-immune adults without malaria

b *P*=0.001 χ^2^

c *P*= 0.0362 Wilcoxon Rank Sum test. Data available from 52 immigrants with malaria and 34 without malaria.

d *P*=0.0890 Wilcoxon Rank Sum test.

### Antibodies in immigrants without malaria

**Figure 1:**
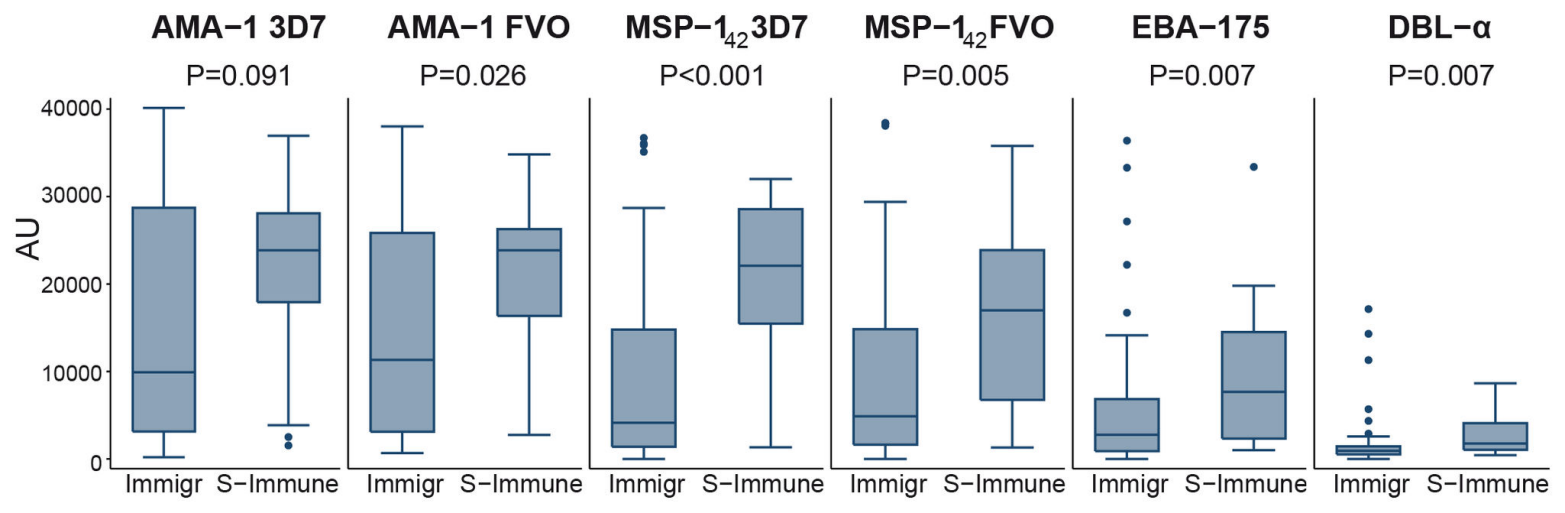
IgG antibody responses to merozoite antigens in immigrants (Immigr) and semi-immune adults (S-Immune) without malaria. Data are presented as boxplots that illustrate the medians and the 25^th^ and 75^th^ quartiles, and the whiskers represent the 10% and 90% percentiles. Outliers are marked with circles. *P*-values were calculated using the Wilcoxon Rank Sum test. Cutoff values for seroprevalences were 238.02 AU for AMA-1 3D7; 1134.73 AU for AMA-1 FVO; 921.18 AU for MSP-1_42_ 3D7; 638.33 AU for MSP-1_42_ FVO; 3110.36 AU for EBA-175; 1572.55 AU for DBL-α.

IgG levels and seroprevalence against erythrocytic antigens associated with immunity, and against a set of IE of different origins, were determined in immigrants and compared to semi-immune adults from Mozambique to assess if the cessation of exposure had an effect on basal Ab levels. IgG levels (Figure 1) and seroprevalences (Table 2) against all recombinant proteins tested were lower in non-infected immigrants compared to healthy semi-immune adults, with the exception of AMA-1 3D7 that the difference between groups was not statistically significant, and the seroprevalence of DBL-α that was similar in both groups.

**Table 2:** IgG seroprevalence in immigrants and semi-immune adults without malaria.

	**Immigrants (n=37)**		**Semi-Immune (n=27)**		
	**n**	**%**	**n**	**%**	*P*-value*
AMA-1 3D7	35	95	27	100	0.504
AMA-1 FVO	30	81	27	100	**0.018**
MSP-1_42_ 3D7	31	84	27	100	**0.035**
MSP-1_42_ FVO	30	81	27	100	**0.018**
EBA-175	12	32	17	63	**0.022**
DBL-α	7	19	12	44	0.051

* Fisher’s exact test

### Antibodies in immigrants upon malaria reinfection

**Figure 2:**
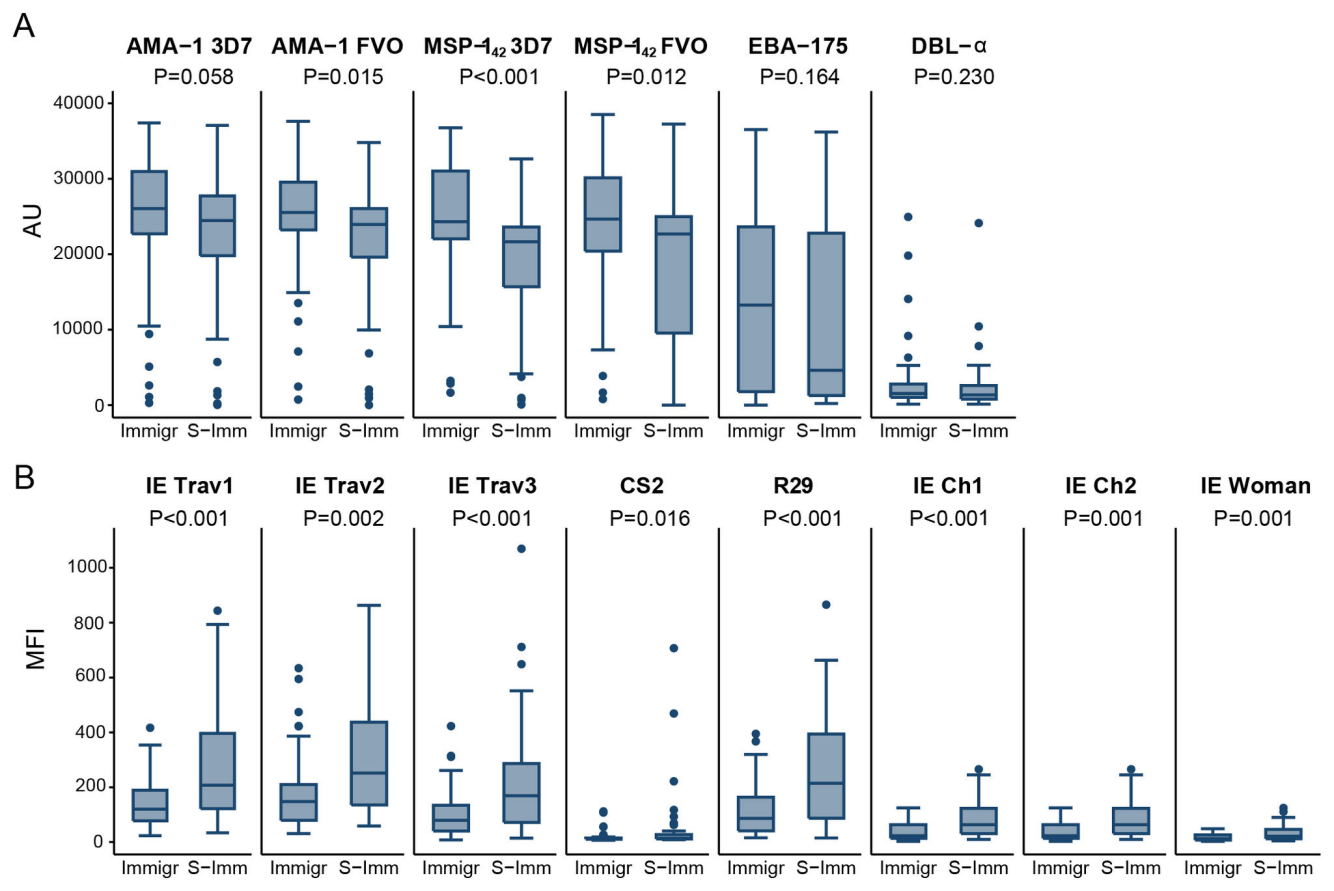
IgG antibody responses to merozoite antigens (A) and *P. falciparum* Infected Erythrocytes (B) in immigrants (Immigr) and Semi-Immune adults (S-Imm) with clinical malaria. Data are presented as boxplots that illustrate the medians and the 25^th^ and 75^th^ quartiles, and the whiskers represent the 10% and 90% percentiles. Outliers are marked with circles. *P*-values were calculated using the Wilcoxon Rank Sum test. Cutoff values for seroprevalences were 238.02 AU for AMA-1 3D7, 1134.73 AU for AMA-1 FVO, 921.18 AU for MSP-1_42_ 3D7, 638.33 AU for MSP-1_42_ FVO,3110.36 AU for EBA-175, 1572.55 AU for DBL-α, 36.88 MFI for IE_Trav1_, 38.55 MFI for IE_Trav2_, 24.75 MFI IE_Trav3,_ 14.23 MFI for CS2, 19.92 MFI for R29, 8.69 MFI for E_Ch1,_ 1.98 MFI for IE_Ch2,_ and 5.54 MFI for IE_Woman_.

Ab levels and prevalence were determined in immigrants and semi-immune adults, both with an acute episode of clinical malaria (Figure 2, Table 3). Immigrants showed higher Ab levels against the merozoite antigens AMA-1 and MSP-1_42_ (Figure 2A). There were no differences in Ab responses to EBA-175 and DBL-α. However, immigrants had significantly lower levels of Ab against all IEs compared to semi-immune adults (Figure 2B). No differences were detected in seroprevalence between immigrants and semi-immune adults with the exception of IE_Ch1_, which was higher in the later (Table 3).

**Table 3:** IgG seroprevalence in immigrants and semi-immune adults with a clinical malaria episode, and naïve adults with a primary infection.

	**Immigrants (n=55)**		**Semi-Immune (n=50)**		**Travelers (n=20)**		*P*-value*	
	**n**	**%**	**n**	**%**	**n**	**%**	**Immigrants vs Semi-Immune**	**Immigrants vs Travelers**
AMA-1 3D7	54	98	48	96	9	45	0.604	**<0.001**
AMA-1 FVO	54	98	47	94	6	30	0.345	**<0.001**
MSP-1_42_ 3D7	55	100	47	94	10	50	0.105	**<0.001**
MSP-1_42_ FVO	54	98	46	92	8	40	0.189	**<0.001**
EBA-175	34	62	25	50	0	0	0.243	**<0.001**
DBL-α	22	40	14	28	3	15	0.222	0.054
IE_Trav1_	51	93	49	98	4	20	0.366	**<0.001**
IE_Trav2_	52	95	50	100	7	35	0.245	**<0.001**
IE_Trav3_	49	89	49	98	1	5	0.115	**<0.001**
CS2	15	27	22	44	0	0	0.102	**0.008**
R29	50	91	48	96	7	35	0.441	**<0.001**
IE_Ch1_	49	89	50	100	1	5	**0.028**	**<0.001**
IE_Woman_	47	85	47	94	1	5	0.207	**<0.001**
IE_Ch2_	50	91	50	100	2	10	0.058	**<0.001**

* Fisher’s exact test

**Figure 3:**
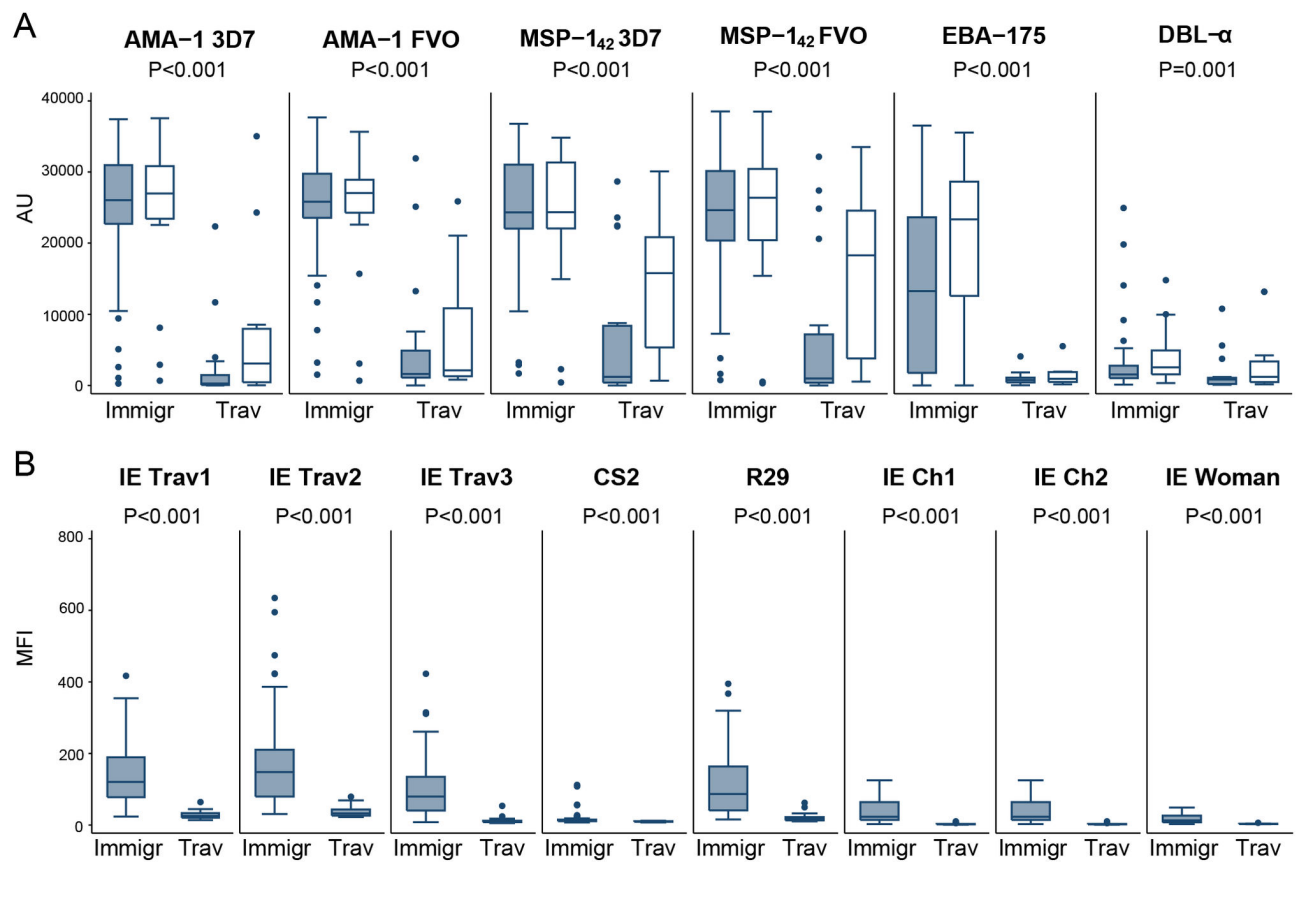
IgG antibody responses to merozoite antigens (A) and *P. falciparum* Infected Erythrocytes (B) in immigrants (Immigr) and travelers (Trav) with clinical malaria. IgG levels were determined at the acute episode of malaria (day 0, black boxes) and at convalescence (day 7, white boxes). Data are presented as boxplots that illustrate the medians and the 25^th^ and 75^th^ quartiles, and the whiskers represent the 10% and 90% percentiles. Outliers are marked with circles. *P*-values were calculated using the Wilcoxon Rank Sum test. Cutoff values for seroprevalences were 238.02 AU for AMA-1 3D7, 1134.73 AU for AMA-1 FVO, 921.18 AU for MSP-1_42_ 3D7, 638.33 AU for MSP-1_42_ FVO,3110.36 AU for EBA-175, 1572.55 AU for DBL-α, 36.88 MFI for IE_Trav1_, 38.55 MFI for IE_Trav2_, 24.75 MFI IE_Trav3,_ 14.23 MFI for CS2, 19.92 MFI for R29, 8.69 MFI for E_Ch1,_ 1.98 MFI for IE_Ch2,_ and 5.54 MFI for IE_Woman_.

Next, Ab responses in immigrants were compared with Ab responses in travelers during an acute malaria episode and in convalescence (Figure 3, Table 3). Overall, infected immigrants had higher Ab levels and seroprevalences against erythrocytic antigens (Figure 3A, Table 3) and IEs (Figure 3B, Table 3) compared to travelers.

### Effect of time since cessation of exposure on antibody levels

The effect of time since immigration on Ab levels in individuals without malaria or with a clinical malaria episode was assessed. None of the Ab against the antigens tested showed any correlation with the time since migration (data not shown) in immigrants with malaria or without malaria. Similarly, no significant differences were found in IgG levels or seroprevalences between individuals that had migrated from their malaria endemic country of origin 5 or fewer years ago, and those that had migrated more than 5 years ago, without malaria (Table S1) or with clinical malaria (Table S2).

## Discussion

In this study we found that after long periods without continuous malaria exposure, immigrants from endemic areas without malaria still presented seropositivities of 32% to 98% for erythrocytic antigens considered as leading vaccine candidates, and immigrants with clinical malaria had similar seroprevalences than semi-immune adults also with a clinical episode However, immigrants without malaria had lower levels of IgGs to all antigens tested compared to healthy adults with continuous life-long exposure to malaria. Although this may suggest that there is some loss of immunity, in this cohort we did not observe an association between time since migration and Ab levels, which may indicate that IgG are longer-lived than usually perceived, at least in adults. In addition, we cannot discard that the healthy adults recruited in Mozambique may have been recently exposed.

Immigrants with clinical malaria had higher IgG levels compared to immigrants without malaria and compared to naïve adults with a primary infection. This may indicate that even at day of presentation to the clinics with malaria, there is boosting of Ab against a wide repertoire of antigens. Rapid boosting of Ab responses to various *P. falciparum* antigens has been reported after re-exposure to malaria following prolonged periods of either sustained control or low transmission, in both children and adults [35,40], suggesting that there is maintenance of immune memory and the capacity to respond quickly to reinfection. Nevertheless, previous exposure could account for part of this difference between the two groups of immigrants. Migrants who contracted malaria upon return to their countries of origin could have been historically more exposed than migrants without clinical malaria (i.e. because there is higher malaria transmission intensity in those areas). However, we think this is unlikely since the subjects were form highly diverse areas. In addition, differences with the group of travelers could be due to different times of presentation to clinical attention: partially immune immigrants could have a smoldering blood stage infection for weeks whereas travelers would require clinical attention within a few days of the start of the blood stage infection, before their antibody response peaked. This, however, argues in favor of our hypothesis that there is maintenance of immunity.

Interestingly, the magnitude of Ab responses in immigrants with clinical malaria compared to Mozambican semi-immune adults with clinical malaria varied according to the type of antigen. Three different response patterns could be identified. First, immigrants had higher levels of IgG against the merozoite antigens AMA-1 and MSP-1_42_, which are relatively polymorphic/dimorphic [11,53] and highly immunogenic, although many studies have associated them with malaria exposure rather than protection [54–56]. In contrast, both study groups had similar levels of Ab against EBA-175 and DBL-α. EBA-175 is more conserved and less immunogenic than AMA-1 and MSP-1_42_, but more consistently associated with protection from clinical malaria [8,54,55] in our previous studies. DBL-α is also less immunogenic and relatively polymorphic considering that it is a *Pf*EMP-1 domain, which is a highly diverse protein [13]. In fact, it is the most conserved *Pf*EMP-1 domain, probably due to its function in mediating rosetting, and Ab against it have been associated with protection against malaria severity [57]. Finally, immigrants had lower levels of IgG against VSA on IEs that are the most polymorphic and poorly immunogenic upon initial exposures to parasite infections [58]. Therefore, these results together with our previous data on Ab responses to these antigens in young Mozambican children [8,52,58] suggest that, after extended periods in the absence of *P. falciparum* exposure, the magnitude of Ab recall responses upon reinfection depend on the nature of the antigen, as does the pattern of acquisition of such responses upon initial parasite exposure. Nevertheless, genetic and/or environmental differences between migrants who were originally from very diverse countries, and semi-immune adults who were from a unique African endemic area, could also account for some of the differences observed between these groups.

The finding that clinical malaria in immigrants induced higher Ab levels for the more immunogenic antigens may be attributable to a B cell response inclined toward merozoite antigens instead of a broad B cell response against a wider repertoire of different antigens. In contrast, the semi-immune group had greater Ab reactivity against multiple variants of IE antigens, indicating that the response under continuous exposure may be directed toward control of parasite variants [12]. Immunity to IEs is slowly acquired in infancy due to the high polymorphism of parasite antigens expressed on the surface of the erythrocytes, resulting in the need of cumulative exposures to acquire an Ab repertoire able to recognize antigenically distinct *Pf*EMP-1 molecules [59,60]. The interruption of malaria exposure in immigrants, and therefore the interruption of acquisition of immunity to VSA, may explain why they have lower Ab levels and why they do not respond to the same extent as continuously exposed semi-immune adults upon re-exposure against these antigens. However, since we do not have data on their antibody levels at the time of migration we cannot discard that the lower levels are due to a loss of immunity.

In contrast, the maintenance of Ab responses against DBL-α and EBA-175, which have been shown to be important in protection from clinical and severe malaria, could represent evidence that immunity to the most severe manifestations of clinical malaria is largely maintained, as clinical data appear to show [14–20]. Data comparing IgG levels in immigrants with malaria against IgG levels in naïve adults with a primary infection represent further evidence of maintenance of immunity, as immigrants had higher IgG levels against all antigens and IEs. This is consistent with clinical observations that migrants rarely get severe/deadly malaria compared to travelers [14–21].

Although our results show that there are *P. falciparum*-specific Ab memory responses after long periods without exposure to malaria, the effect of these responses on immunity is not clear. We do not know if these Ab responses would lead to protection from infection or clinical/severe malaria upon reinfection. However, immigrants returning with clinical malaria have not been protected from infection or clinical malaria in this occasion, but this does not imply per se that they have no immunity to malaria. It could be that the progression of the disease was different than the naïve or the semi-immune adults. Unfortunately, we do not have data on the progression of the disease. In addition, we cannot conclude that they have lost immunity since even continuously exposed adults may have clinical malaria, which is what we observed also with the group of semi-immune adults in Mozambique. We are probably facing two different layers of immunity, in which the maintenance of basal levels of Ab is key in the initial control of a reinfection, whereas the capacity to generate effector responses (new Ab and MBC) may lead to prevention of malaria pathogenicity and severity.

Previous findings on long-lived malaria-specific MBC support maintenance of memory responses. Malaria-specific MBC were detected in adults from a low endemicity setting in Thailand and found to persist for more than 7 years without ongoing exposure [44]. In The Gambia, the median number of malaria-specific MBC was similar to the median number of diphtheria-specific MBC, suggesting that the malaria-specific circulating MBC pool is of similar magnitude to that of other antigens [45]. However, it is not clear whether Ab levels correlate with MBC, and the maintenance of the plasma cell pool may be independent of MBC [61]. *P. falciparum* Ab and MBC have been shown to correlate when there is recent exposure, but correlation does not seem to persist after long periods of non-exposure [32,39,42,45]. The role and contribution of MBC in Ab levels of a recall response during a malaria infection needs to be addressed more thoroughly.

While Ab responses to erythrocytic antigens seem to be maintained to a large extent, cytokine profiling in these same individuals showed that, upon loss of exposure, control of pro-inflammatory response and tolerance to *P. falciparum* may be reduced [62]. No correlation was found between plasma cytokine or chemokine concentrations and Ab levels. However, other Ab responses involved in “anti-disease immunity” or tolerance [63] may be directly implicated in the cellular immune response, and may be more short-lived than the Ab responses measured in this study.

The results of this work highlight the usefulness of migrant populations’ studies to understand the maintenance of immunity to malaria and to disentangle determinants and mediators, allowing the study of memory immune responses without the interference of natural malaria exposure. Nevertheless, several limitations were faced. We could not control for the immune status of the migrants when arriving to the non-endemic area, or for the number of returns to their country of origin with possible malaria exposure and boost of the immune responses. However, none of them had a malaria episode after their migration and the exposure may have been minimal due to the few times they returned and the nature of these short trips. Longitudinal migrant cohort studies would resolve the limitations of the present study and would allow for more precise assessment of long-term immunological maintenance. Further research into the factors that determine the duration of specific Ab responses and the underlying immune mechanisms will be important for vaccine design to induce levels of immunity that are more protective and sustained than those attained by current vaccine candidates [64]. Public health will ultimately benefit from a better understanding of the mechanisms involved in the maintenance of immunity upon loss of exposure, since malaria control strategies leading to diminished malaria transmission may decrease the population’s immunity with detrimental consequences in the case of malaria resurgence.

## Supporting Information

Figure S1 **Luminex IgG curves for each tested antigen made with a pool of plasma samples from hyper-immune Mozambican adult volunteers.** Data is representative from one experiment in duplicates.
(DOCX)

Figure S2 **Representative flow cytometry data of antibodies to IEs surface antigens assay.** Panels A, B, and C show the dot plots for the lab parasite R29 and panels D, F and G the dot plots for one field isolate (D, F, G). Samples tested were the pool of plasma samples from hyper-immune Mozambican adult volunteers (A, D), the pool from non-exposed European adults (B, E) and plasma from one migrant patient (C, F).
(DOCX)

Table S1 **Plasma IgG levels and seroprevalence in immigrants without malaria who have been ≤ 5 years or > 5 years in a non-endemic area.**
(DOCX)

Table S2 **Plasma IgG levels and seroprevalence in immigrants with a clinical a malaria episode who have been ≤ 5 years or > 5 years in a non-endemic area.**
(DOCX)
