# Supplementary figures and images for: High Antibody Responses against Plasmodium falciparum in Immigrants after Extended Periods of Interrupted Exposure to Malaria
Source: PLoS One. 2013 Aug 14;8(8):e73624. doi: 10.1371/journal.pone.0073624 (PMC3743903; doi:10.1371/journal.pone.0073624)

**Figure S1**


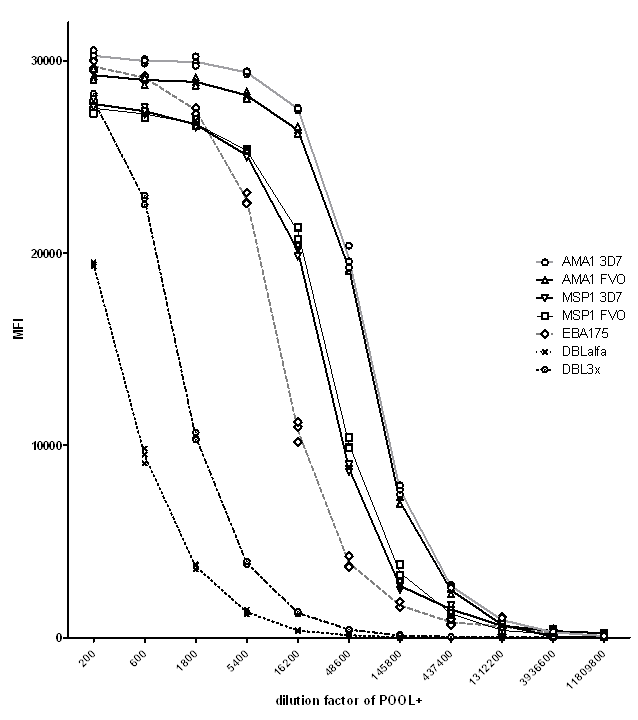

Supplement: Figure S1 — Luminex IgG curves for each tested antigen made with a pool of plasma samples from hyper-immune Mozambican adult volunteers. Data is representative from one experiment in duplicates. (DOCX) [file pone.0073624.s001.docx]

**Figure S2**

**
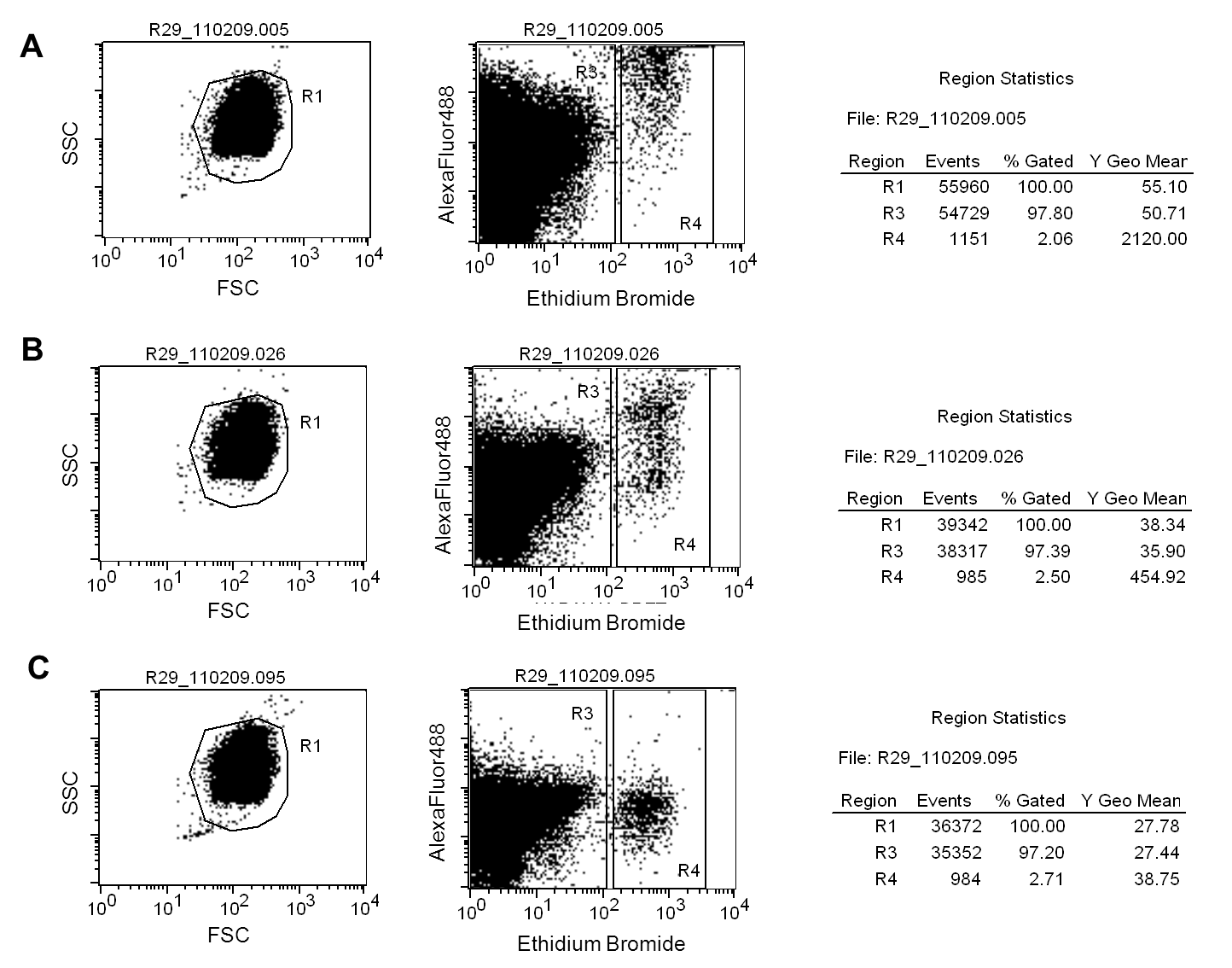
**

**
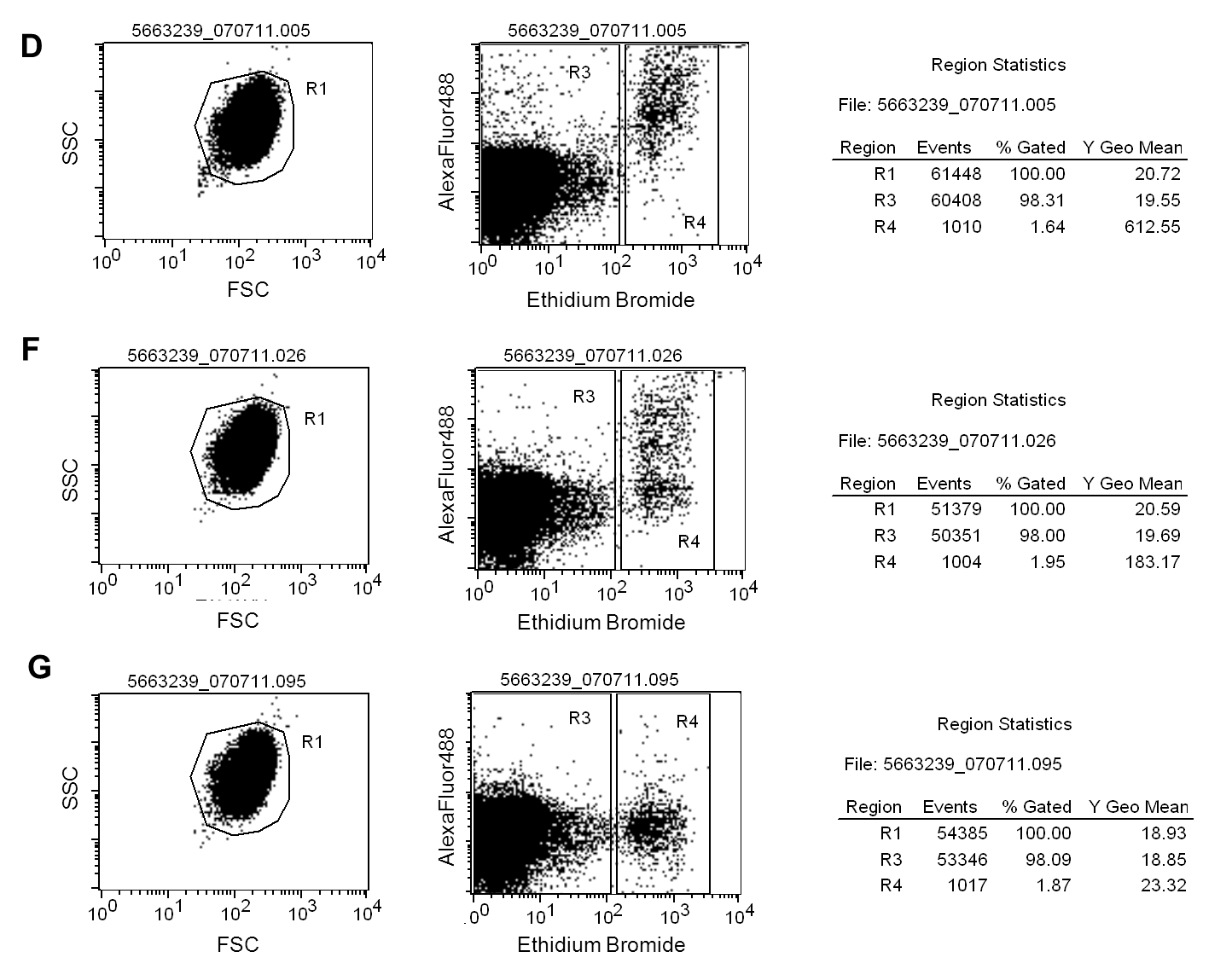
**

Supplement: Figure S2 — Representative flow cytometry data of antibodies to IEs surface antigens assay. Panels A, B, and C show the dot plots for the lab parasite R29 and panels D, F and G the dot plots for one field isolate (D, F, G). Samples tested were the pool of plasma samples from hyper-immune Mozambican adult volunteers (A, D), the pool from non-exposed European adults (B, E) and plasma from one migrant patient (C, F). (DOCX) [file pone.0073624.s002.docx]
