# Supplementary material for: High Antibody Responses against Plasmodium falciparum in Immigrants after Extended Periods of Interrupted Exposure to Malaria
Source: PLoS One. 2013 Aug 14;8(8):e73624. doi: 10.1371/journal.pone.0073624 (PMC3743903; doi:10.1371/journal.pone.0073624)
Supplement: Table S1 — Plasma IgG levels and seroprevalence in immigrants without malaria who have been ≤ 5 years or > 5 years in a non-endemic area. (DOCX) [file pone.0073624.s003.docx]

**Table S1.** Plasma IgG levels and seroprevalence in immigrants without malaria who have been ≤ 5 years or > 5 years in a non-endemic area.

|  | Immigrants without malaria | | | | | | | | | |
| --- | --- | --- | --- | --- | --- | --- | --- | --- | --- | --- |
|  | ≤ 5 years (n=17) | | > 5 years (n=17) | |  | ≤ 5 years | | > 5 years | |  |
|  | Median, AU | (IQR) | Median, AU | (IQR) | *P*-value* | n | % | n | % | *P*-value** |
| AMA-1 3D7 | 14892.1 | (4478.25; 36224.40) | 4355.29 | (3130.65; 21031.20) | 0.196 | 15 | 88 | 17 | 100 | 0.485 |
| AMA-1 FVO | 13675.5 | (1983.34; 34070.10) | 5534.9 | (3630.35; 20307.70) | 0.380 | 13 | 76 | 15 | 88 | 0.656 |
| MSP-1**_42_** 3D7 | 3042.69 | (1100.35; 25723.50) | 4707.45 | (1867.79; 8601.08) | 0.850 | 13 | 76 | 15 | 88 | 0.656 |
| MSP-1**_42_** FVO | 3562.79 | (2109.54; 22207.00) | 6601.09 | (1233.23; 13086.00) | 0.570 | 14 | 82 | 14 | 82 | 1.000 |
| EBA-175 | 1888.38 | (118.68; 6526.38) | 2516.26 | (855.73; 7059.46) | 0.524 | 6 | 35 | 6 | 35 | 1.000 |
| DBL-α | 777.35 | (341.57; 1430.87) | 1203.66 | (660.10; 1928.82) | 0.102 | 3 | 18 | 4 | 24 | 1.000 |

* Wilcoxon Rank Sum test; **Fisher's exact test
