# Supplementary material for: High Antibody Responses against Plasmodium falciparum in Immigrants after Extended Periods of Interrupted Exposure to Malaria
Source: PLoS One. 2013 Aug 14;8(8):e73624. doi: 10.1371/journal.pone.0073624 (PMC3743903; doi:10.1371/journal.pone.0073624)
Supplement: Table S2 — Plasma IgG levels and seroprevalence in immigrants with a clinical a malaria episode who have been ≤ 5 years or > 5 years in a non-endemic area. (DOCX) [file pone.0073624.s004.docx]

**Table S2.** Plasma IgG levels and seroprevalence in immigrants with a clinical a malaria episode who have been ≤ 5 years or > 5 years in a non-endemic area.

|  | Immigrants with malaria | | | | | | | | | |
| --- | --- | --- | --- | --- | --- | --- | --- | --- | --- | --- |
|  | ≤ 5 years (n=20) | | > 5 years (n=32) | |  | ≤ 5 years | | > 5 years | |  |
|  | Median | (IQR) | Median | (IQR) | *P*-value* | n | % | n | % | *P*-value** |
| Recombinant proteins (AU) | | | | | | | | | | |
| AMA-1 3D7 | 25377.1 | (23061.70; 29775.85) | 27100.3 | (23416.55; 31606.35) | 0.735 | 20 | 100 | 31 | 97 | 1.000 |
| AMA-1 FVO | 26189.9 | (23450.40; 31323.25) | 25401.05 | (23871.10; 28845.15) | 0.645 | 20 | 100 | 31 | 97 | 1.000 |
| MSP-1**_42_** 3D7 | 25105.5 | (22491.50; 31200.50) | 24313.4 | (22167.75; 31620.70) | 0.652 | 20 | 100 | 32 | 100 | . |
| MSP-1**_42_** FVO | 26662 | (20281.75; 30515.15) | 24825.9 | (20398.60; 29305.70) | 0.880 | 20 | 100 | 32 | 100 | . |
| EBA-175 | 4269.41 | (1664.27; 22227.55) | 19134.9 | (2906.40; 24067.80) | 0.522 | 12 | 60 | 22 | 69 | 0.561 |
| DBL-α | 1780.92 | (1030.28; 2335.96) | 1557.16 | (1062.31; 3017.56) | 0.778 | 9 | 45 | 13 | 41 | 0.780 |
| IEs (MFI) | | | | | | | | | | |
| IE_Trav1_ | 148.99 | (75.61; 228.48) | 117.88 | (77.71; 165.95) | 0.652 | 17 | 85 | 31 | 97 | 0.285 |
| IE_Trav2_ | 168.42 | (65.78; 252.01) | 140.59 | (89.85; 198.10) | 0.535 | 18 | 90 | 31 | 97 | 0.551 |
| IE_Trav3_ | 111.43 | (29.87; 211.92) | 71.24 | (42.04; 102.51) | 0.357 | 15 | 75 | 31 | 97 | 0.026 |
| CS2 | 12.66 | (11.69; 15.92) | 12.47 | (10.28; 15.03) | 0.742 | 5 | 25 | 10 | 31 | 0.757 |
| R29 | 81.2 | (37.58; 212.52) | 92.47 | (41.42; 153.80) | 0.721 | 18 | 90 | 29 | 91 | 1.000 |
| IE_Ch1_ | 39.69 | (10.04; 76.38) | 22.65 | (15.77; 56.12) | 0.625 | 15 | 75 | 31 | 97 | 0.026 |
| IE_Woman_ | 15.56 | (6.02; 30.05) | 11.16 | (7.77; 24.52) | 0.510 | 16 | 80 | 28 | 88 | 0.695 |
| IE_Ch2_ | 25.83 | (4.41; 50.68) | 18.91 | (9.33; 33.67) | 0.821 | 16 | 80 | 31 | 97 | 0.066 |

* Wilcoxon Rank Sum test; **Fisher's exact test
